# Do synbiotics really enhance beneficial synbiotics effect on defecation symptoms in healthy adults? Randomized, double-blind, placebo-controlled trial

**DOI:** 10.1097/MD.0000000000028858

**Published:** 2022-02-25

**Authors:** Daisaku Ito, Yuta Yamamoto, Takao Maekita, Naoko Yamagishi, Shuji Kawashima, Takanori Yoshikawa, Kensuke Tanioka, Takeichi Yoshida, Mikitaka Iguchi, Kosei Kunitatsu, Yoshimitsu Kanai, Seiya Kato, Masayuki Kitano

**Affiliations:** a Second Department of Internal Medicine, Wakayama Medical University, Kimiidera 811-1 Wakayama, Japan; b Department of Anatomy and Cell Biology, Wakayama Medical University, Kimiidera 811-1 Wakayama, Japan; c Department of Emergency and Critical Care Medicine, Wakayama Medical University, Kimiidera 811-1 Wakayama, Japan; d Clinical Study Support Center, Wakayama Medical University, Kimiidera 811-1 Wakayama, Japan.

**Keywords:** BDHQ, *Bifidobacterium longum* NT strain, PAC-SYM, probiotics, synbiotics

## Abstract

**Goals:**

We examined whether synbiotics enhance improvement by probiotics.

**Background:**

Probiotics, which are beneficial microbacteria, are a nutritional intervention for treatment of functional constipation or its tendency. Prebiotics, meanwhile, can promote the proliferation of probiotics in the gastrointestinal tract and enhance their beneficial effects. Synbiotics, a combination of probiotics and prebiotics, may be superior to probiotics in the treatment of defecation-related symptoms, but this requires elucidation.

**Study:**

This randomized, double-blind, placebo-controlled study enrolled 69 healthy adults with constipation tendency. Participants were allocated to either control, probiotics, or synbiotics groups and they recorded details of their defecations and their condition. The first 2 weeks were the observation period and the latter 2 weeks were the intervention period, in which participants took test foods. Probiotic foods included *Bifidobacterium longum* NT strain (10^10^ CFU/day), synbiotic foods included the NT strain (10^10^ CFU/day) and galactooligosaccharide (1 g/day). Placebo foods contained the vehicle only. Participants answered questionnaires (*Patient Assessment on Constipation Symptoms* [PAC-SYM], and one on dietary history) on the last day of each period.

**Results:**

Nine participants withdrew consent, and 2 of the remaining 60 had missing data. Age, body mass index, and sex were not significantly different between the 3 groups. Frequency of bowel movements in the fourth week, the primary endpoint, was not increased in the probiotics or synbiotics groups compared with the control group, and the frequency of bowel movements and days with defecation were not changed by probiotics or synbiotics during the intervention period. Probiotics and synbiotics did not improve stool conditions, although incomplete defecation was improved by probiotics but not by synbiotics compared with placebo. PAC-SYM indicated that stool condition and total scores were improved by probiotics but not by synbiotics during the intervention compared with placebo.

**Conclusion:**

The probiotic strain *Bifidobacterium longum NT* can improve constipation symptoms, especially stool condition, but it does not increase bowel movement frequency in healthy adults with constipation tendency. Synbiotics treatment seemed to diminish this improvement of constipation induced by probiotics. This study indicates the possibility of attenuation of beneficial effects from probiotics by the use of synbiotics, contrary to synbiotics theory.

## 1 Introduction

Functional constipation is a common health problem in clinical practice, especially in Asian countries, where it is reported in 27% of patients, compared with 2% reported in North America.^[1,2]^ Rome IV criteria for the diagnosis of functional constipation focuses on the frequency of spontaneous bowel movement and difficulty in defecation (e.g., straining, hard stools, incomplete evacuation and manual maneuvers to facilitate defecation).^[3]^ Functional constipation is not directly life-threatening, but symptoms of chronic constipation have been reported to negatively affect quality of life and can affect increase in economic loss, including by increased work absenteeism.^[3–5]^ Improving or preventing functional constipation is therefore important. Constipation symptoms can be improved by medical treatments, specifically laxatives. Meanwhile, constipation tendency can often be prevented by nutritional treatments.^[6]^

Probiotics, microorganisms indicated to have beneficial effects for humans, are part of some fermented foods and are often used in nutritional treatment of intestinal diseases including constipation.^[7]^ Recent meta-analysis indicated that probiotics treatment for more than 14 days also increased stool frequency in healthy adults and in patients with functional constipation.^[8]^ The beneficial effect was shown to be induced by certain specific strains of microorganisms, but not by all probiotics.^[9]^ Clinical studies are therefore needed to confirm whether a specific strain has any beneficial effect.

Prebiotics are non-digestible foods that affect health by stimulating the growth and/or activity of colonic *Bifidobacteria.*^[10]^ Galactooligosaccharide as a prebiotic treatment has been reported to increase *Bifidobacteria* in human fecal microflora,^[11,12]^ and to increase the frequency of bowel movement in pediatric patients and in healthy adult volunteers with tendency for constipation.^[13]^ Synbiotics, meanwhile, are the combination of probiotics and prebiotics. Beneficial effects of prebiotics are thought to include promotion of the growth of probiotics in the colon. The efficacy of synbiotics on the increase of bowel movement frequency was substantiated by a randomized double-blind placebo-controlled trial.^[14]^ Whether synbiotics are superior to probiotics alone for improvement of bowel movement frequency still requires validation.

We therefore primarily examined whether probiotics increase bowel movement frequency in otherwise healthy adults with tendency for constipation. We also examined whether synbiotics further increased bowel movement frequency compared with probiotics. The changes of constipation symptoms induced by probiotics and synbiotics were analyzed in order to examine the mechanisms of improving bowel movement frequency in probiotics and synbiotics.

## 2 Materials and methods

### 2.1 Study design

This 4-week trial was performed using a double-blind, placebo-controlled, parallel group design. Baseline was observed in the first 2 weeks (observation period), and the effect of intervention was observed in the latter 2 weeks (intervention period). For the entire trial, the participants were asked to record the dates and times of every defecation, the consistency of their stools according to the Bristol stool scale (See Table S1, Supplemental Digital Content),^[15]^ the degree of straining, any pain after defecation, and if they had feeling of incomplete evacuation (1: absent, 2: mild, 3: moderate, 4: severe, and 5: very severe). Participants were asked to take 2 capsules each morning and evening in the intervention period. They answered 2 questionnaires on days 14 and 28, the final days of each period. The first questionnaire was *Patient Assessment on Constipation Symptoms* (PAC-SYM), which was used to assess the symptoms of constipation over the latter 2 weeks. This was followed by *Brief Diet History Questionnaire* (BDHQ), used to assess the dietary history. PAC-SYM consists of 12 questions on abdominal, rectal and stool symptoms within the previous 2-week period (See, Table S2, Supplemental Digital Content), and is used to assess the symptoms of constipation.^[16]^ BDHQ was based on a self-administered dietary history questionnaire.^[17,18]^

Before the start of this study, test foods, questionnaires, and defecation records were sent to participants in late January 2019. Participants were asked to start the trial by February 2019 and to return the answered questionnaires and defecation records after completing the trial period. The study procedures were approved by the Wakayama Medical University Ethics Committee (approval number 2325) and registered as a University Hospital Medical Information Network Clinical Trials Registry clinical trial (unique trial number: UMIN000033185).

### 2.2 Participants

This study evaluated the increase of bowel movement frequency in probiotic or symbiotic foods. This study excluded patients with chronic constipation meeting the Rome IV criteria because such patients may use laxatives, which affect bowel movement frequency for as long as 4 weeks, this study period. Previous studies recruited healthy men and women with constipation tendency.^[19,20]^ This study thus also recruited healthy adult men and women (20–60 years of age) who had bowel movement frequency of just 2 or 3 movements per week from Wakayama and Osaka prefectures in Japan, and their eligibility was assessed by doctors.

The following exclusion criteria were used:

1. Diagnosis of constipation or fitting Rome IV criteria in the initial interview.
2. History of organic disease in the gastrointestinal tract (participants did not undergo colorectal cancer screening within the previous year, or had been previously diagnosed with organic disease in the gastrointestinal tract).
3. Diagnosed kidney disease, diabetes, hypothyroidism, inflammatory disease, neurological, or psychiatric disease.
4. Subjects who had taken antibiotics or probiotics within the past month.
5. Subjects taking drugs that induce chronic constipation (anticholinergic drugs, psychotropic drugs, antiparkinsonian drugs, opioids, chemotherapeutic drugs, calcium channel blockers, diuretic drugs, antacid, iron preparations, adsorbents, antiemetic drugs, and antidiarrheal drugs).
6. Subjects using biological drugs or steroids, routinely consuming foods containing lactic acid bacteria or bifidus bacteria, or consuming health foods for improvement of constipation.
7. History of allergy to foods containing *Lactobacillus* or bifidus bacteria.
8. Participation in other clinical tests concerning food containing probiotics within one month.
9. Subjects whose use of constipation drugs or consumption of foods for constipation improvement cannot be stopped during the research period.
10. Subjects who will travel overseas during the research period.
11. Subjects judged to be otherwise unsuitable for participation in the trial.

### 2.3 Sample size

Sample size was computed based on the primary end-point measure of bowel movement frequency within the second week of the period of when test food was taken. In a previous report, bowel movement frequencies were 4.1 ± 1.7 per week in a *Bifidobacterium lactis* DN-173010 strain group and 2.6 ± 1.0 per week in a placebo group in the second week of the period when test food was taken.^[21]^ According to these results, 21 subjects per group were needed to achieve statistical power of 0.80 with a type I error of 0.05 for comparison of the bowel movement frequency between placebo and probiotics groups 2 weeks after administration of probiotics. Assuming that 10% of subjects would be excluded due to consent withdrawal or by medication affecting bowel movement frequency, we planned enrolment of 23 subjects per group, making 69 subjects in total.

### 2.4 Randomization and masking

After obtaining written informed consent, height and body weight were measured, and age and sex were collected. When the number of eligible participants reached 69, these participants were enrolled. EPIC-Oxford, a cohort study, indicated that the frequency of bowel movement was associated with body mass index (BMI) in both males and females, and with age in females.^[22]^ Adjustment of subject background (age or BMI) may therefore be needed to confirm the efficacy of probiotics.

In this study, a static allocation table adjusting BMI (<21.75/ ≥21.75) and age (20–40/41–60) was made by the Clinical Study Support Center at Wakayama Medical University Hospital. Participant lists including test ID, BMI and sex were sent to assignment staff in January 2019, who performed the allocation according to the table. The allocation results were then sent to a facilitator, who labeled the boxes containing test foods according to the results of allocation with ID for testing purposes. The individuals related to the allocation were not connected to the research staff, and all research staff and enrolled participants were unaware of the actual allocations. All data were fixed on 6 November 2019, and the allocation key opening was performed. Data were analyzed by 2 statisticians at the Clinical Study Support Center at Wakayama Medical University, according to the statistical analysis plan.

### 2.5 Test foods

The test foods were capsules containing starch, calcium stearate and microcrystalline cellulose. Probiotic capsules also contained the viable cell count of *Bifidobacterium longum* NT strain (2.5 × 10^9^ colony-forming units (cfu)/capsule). Synbiotic capsules also contained the viable cell count of /*Bifidobacterium longum* NT strain (2.5 × 10^9^ cfu/capsule) and galactooligosaccharide (250 mg/capsule). All capsules were made from hydroxypropyl methylcellulose, and all test foods were produced by Genuine R&D (Fukuoka, Japan) according to good manufacturing practice. *Bifidobacterium longum* NT strain was supplied by Noster Inc. (Kyoto, Japan).

### 2.6 Statistical analysis

Primary outcome of this study was bowel movement frequency in the fourth week. Secondary outcomes were bowel movement frequency in the first, second and third weeks, difference of bowel movement frequency or days with defecation between the observation and intervention periods, difference in Bristol stool form scale between the observation and intervention periods, symptoms related to constipation (straining, pain and feeling of incompletion) in defecation, difference of PAC-SYM scores (abdominal, rectal, stool condition, and total) between observation and intervention periods, and difference in nutritional intake between “responders” and “non-responders.” Responders were defined as patients whose bowel movement frequency increased by 2 or more in the intervention period compared with in the observation period. Data were analyzed using SAS 9.4 software (SAS Institute, Cary, NC) for the primary and secondary outcomes, and JMP Pro 14.1.0 software (SAS Institute) for the patient backgrounds (age and BMI). To confirm a static allocation table adjusting BMI and age, one-way ANOVA was performed to compare the subject backgrounds between the 3 groups (age, height, weight, and BMI). Fisher exact test was performed to compare the male/female ratio among the 3 groups. Tukey-HSD test was performed to compare the bowel movement frequency of each week. Dunnett test was performed to compare the degree of straining, pain and incomplete evacuation in defecation, Bristol stool form scale number, and the difference of score in PAC-SYM between probiotics and synbiotics groups against the placebo group. The difference of nutrition intake between responders and non-responders was compared with Student *t* test. Outcomes and their statistical methods were listed (Table S3, Supplemental Digital Content). *P* < .05 was considered to be statistically significant.

## 3 Results

### 3.1 Participants

**Figure 1:**
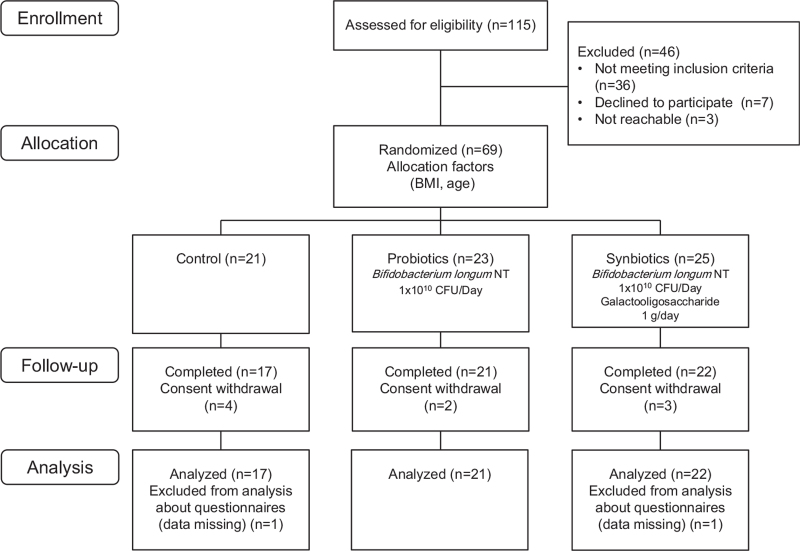
Study flow diagram.

Participant recruitment for this study was between July 1, 2018 and December 26, 2018. Following explanation of the study, 115 people consented to inclusion. Forty six people did not meet inclusion criteria, 7 people declined further participation, and 3 people were unreachable before the start of this study. Sixty nine participants were therefore enrolled, and they were allocated to either control (n = 21), probiotics (n = 23), or synbiotics groups (n = 25). Nine participants withdrew before the study ended, so 60 participants were finally analyzed (Fig. 1). PAQ-SYM and BDHQ questionnaires were missing due to postal non-arrival in the case of 2 participants, so the analysis of PAC-SYM comprised the remaining fifty-eight participants. The average ages, BMI and male/female ratio were not significantly different between the 3 groups (Table 1).

**Table 1:** Participant backgrounds.

	Control	Probiotics	Synbiotics	*P* value
n	17	21	22	
Age	39.4 ± 8.9	36.6 ± 11.3	39.3 ± 8.7	.58
Height	159.5 ± 6.9	161.9 ± 6.9	160.1 ± 6.3	.50
Weight	55.6 ± 10.1	57.2 ± 12.2	55.5 ± 9.1	.84
BMI	21.8 ± 3.6	21.7 ± 3.4	21.6 ± 2.8	.99
Sex (female %)	94.1%	81.0%	90.9%	.53

Data are present as mean ± standard deviation or number (%). BMI = body mass index.

### 3.2 Bowel movement frequency

We analyzed the primary outcome, bowel movement frequency in the fourth week, between 2 out of the 3 groups. Mean bowel movement frequency in the fourth week was 5.4 in the control group (95%CI: 4.2, 6.5), 5.5 in the probiotics group (95%CI: 4.7, 6.2), and 4.3 in the synbiotics group (95%CI: 3.3, 5.4). Mean bowel movement frequency in the fourth week did not differ in the probiotics or synbiotics groups compared with the control group. In first, second and third weeks, there were also no significant differences in bowel movement frequency between 2 out of the 3 groups (Table 2).

**Table 2:** Difference of bowel movement frequency before and after intervention.

	Control		Probiotics		Synbiotics		*P* value		
	Mean	95% CI	Mean	95% CI	Mean	95% CI	Control vs probiotics	Control vs synbiotics	Probiotics vs synbiotics
1st week	4.2	(3.4, 5.0)	4.6	(3.8, 5.4)	3.9	(3.3, 4.5)	.70	.88	.36
2nd week	4.4	(3.5, 5.2)	3.4	(2.8, 4.1)	4.0	(3.4, 4.5)	.15	.69	.49
3rd week	5.1	(3.8, 6.5)	5.2	(4.2, 6.2)	4.5	(3.5, 5.5)	.99	.73	.61
4th week	5.4	(4.2, 6.5)	5.5	(4.7, 6.2)	4.3	(3.3, 5.4)	.98	.33	.21

*P* values were calculated with Tukey-HSD method. CI = confidence interval.

### 3.3 Constipation symptoms

To examine whether probiotics and synbiotics simply increased frequency of bowel movement or whether they also improved daily defecation, we analyzed the difference in frequency of bowel movement between the observation period and the intervention period. Moreover, we also analyzed the difference in the number of days with defecation to focus on decrease of days without defecation in response to consumption of probiotic or symbiotic foods (Table 3). Neither probiotics nor synbiotics groups differed significantly from the control group.

**Table 3:** Difference in defecation behavior between the observation and intervention periods.

	Control		Probiotics		Synbiotics		*P* value	
	Mean	95% CI	Mean	95% CI	Mean	95% CI	Probiotics	Synbiotics
Bowel movement frequency	2.1	(0.7, 3.5)	2.7	(1.7, 3.6)	1.0	(−0.8, 2.7)	.82	.44
Number of days with defecation	1.1	(0.1, 2.0)	1.4	(0.6, 2.3)	−0.2	(−1.3, 0.9)	.83	.16

*P* values were calculated with Dunnett test. CI = confidence interval.

The effects of probiotics and synbiotics on stool consistency were determined using Bristol stool scale scores. The difference in average of Bristol stool scale score between the observation period and intervention period was 0.1 in the control group (95%CI: −0.3, 0.4). Meanwhile, this difference was 0.3 in the probiotics group (95%CI: −0.1, 0.7), and it was 0.3 in the synbiotics group (95%CI: 0.0, 0.5). Dunnett test indicated that consistency of stools was not significantly improved in the probiotics and synbiotics groups compared with the control group.

To evaluate symptoms related to constipation, we analyzed the improvements to straining, pain, and incomplete evacuation in defecation (Table 4). The degree of straining and pain in defecation in the probiotics and synbiotics groups did not differ from the control group between the intervention and observation periods. Meanwhile, the degree of complete defecation was statistically improved in the probiotics group (*P* = .04) and tended to be improved in the synbiotics group (*P* = .08) compared with the control group.

**Table 4:** Difference in feeling discomfort at defecation between the observation and intervention periods.

	Control		Probiotics		Synbiotics		*P* value (vs control)	
	Mean	95% CI	Mean	95% CI	Mean	95% CI	Probiotics	Synbiotics
Straining	−0.1	(−0.6, 0.1)	−0.2	(−0.5, 0.1)	−0.2	(−0.4, 0.0)	.84	.87
Painful	−0.1	(−0.2, 0.0)	−0.1	(−0.4, 0.2)	0.1	(−0.1, 0.2)	.87	.54
Incomplete defecation	0.2	(−0.1, 0.4)	−0.3	(−0.5, 0.0)	−0.2	(−0.4, 0.0)	.04	.08

*P* values were calculated with Dunnett test. CI = confidence interval. Subjects recorded the degree of straining, any pain after defecation, and incidence of incomplete evacuation (1: absent, 2: mild, 3: moderate, 4: severe, and 5: very severe).

To evaluate symptoms related to constipation in the observation and intervention periods, the questionnaire about constipation, PAC-SYM, was answered on the final days of the observation and intervention periods. Total score in PAC-SYM was significantly decreased in the probiotics group compared with the control group (Table 5). The difference in stool symptom scores was also significantly decreased in the probiotics group compared with the control group (Table 5). Stool hardness scores were significantly decreased in the probiotics group compared with the control group. In the synbiotics group, there was a decrease in mean total and stool symptom scores to approximately half of the scores in the probiotics group. There was no improvement of abdominal or rectal symptoms in either the probiotics or synbiotics groups.

**Table 5:** Difference in PAC-SYM scores before and after intervention.

	Control		Probiotics		Synbiotics		*P* value (vs control)	
	Mean	95% CI	Mean	95% CI	Mean	95% CI	Probiotics	Synbiotics
Abdominal symptoms (total of below questions)	−0.2	(−0.9, 0.5)	−1.2	(−2.4, 0.1)	−0.7	(−1.3, −0.1)	.25	.66
Discomfort in abdomen	0.1	(−0.3, 0.6)	−0.2	(−0.7, 0.3)	−0.2	(−0.6, 0.2)	.51	.51
Pain in abdomen	−0.2	(−0.5, 0.1)	−0.3	(−0.8, 0.1)	0.0	(−0.2, 0.3)	.79	.56
Bloating in abdomen	−0.1	(−0.6, 0.3)	−0.6	(−1.1, −0.2)	−0.6	(−0.8, −0.3)	.16	.22
Stomach cramps	0.0	(−0.3, 0.3)	0.0	(−0.4, 0.3)	0.0	(−0.2, 0.2)	.97	1.00
Rectal symptoms (total of below questions)	−0.3	(−0.7, 0.2)	−0.7	(−1.5, 0.1)	−0.3	(−0.9, 0.2)	.58	.98
Painful bowel movement	−0.2	(−0.5, 0.1)	−0.4	(−0.9, 0.1)	0.0	(−0.3, 0.4)	.64	.65
Rectal burning during or after bowel movement	−0.1	(−0.2, 0.1)	−0.1	(−0.3, 0.1)	0.0	(−0.1, 0.0)	.93	.99
Rectal bleeding or tearing during or after bowel movement	0.0	(−0.3, 0.3)	−0.1	(−0.5, 0.2)	−0.3	(−0.6, −0.1)	.74	.24
Stool symptoms (total of below questions)	0.3	(−1.5, 2.2)	−2.6	(−4.3, −1.0)	−1.5	(−2.6, −0.4)	.02	.20
Incomplete bowel movement	−0.1	(−0.4, 0.3)	−0.6	(−1.0, −0.3)	−0.3	(−0.7, 0.0)	.08	.50
Bowel movement too hard	0.1	(−0.4, 0.6)	−0.8	(−1.3, −0.3)	−0.2	(−0.6, 0.2)	.02	.66
Bowel movement too small	0.3	(0.0, 0.5)	−0.4	(−0.8, 0.2)	−0.2	(−0.5, 0.2)	.12	.23
Straining or squeezing to pass bowel movement	0.1	(−0.4, 0.5)	−0.5	(−0.9, 0.0)	−0.5	(−1.0, −0.1)	.20	.15
Feeling like had to pass bowel movement but could not	0.0	(−0.5, 0.5)	−0.4	(−0.8, −0.1)	−0.2	(−0.6, 0.1)	.25	.62
Total of 12 questions	−0.1	(−2.1, 1.9)	−4.5	(−7.7, −1.2)	−2.1	(−3.7, −0.6)	.04	.43

*P* values were calculated with Dunnett test. CI = confidence interval, PAC-SYM = Patient Assessment of Constipation Symptoms.

To explore the effect of nutrition intake on bowel movement, we analyzed the difference in nutrition intake between responders and non-responders (Table 6). In the control group, intake of calories, food volume, and fiber tended to be increased in responders compared with non-responders, and water intake also tended to be increased. In the probiotics group, intake of the compositions did not differ between responders and non-responders. In the synbiotics group, calorie and lipid intakes were significantly increased in responders compared with non-responders. No adverse events occurred during this study.

**Table 6:** Difference in intake of nutrition between responders and non-responders.

	Control		Probiotics		Synbiotics		*P* value (responders vs non-responders)		
	Mean	95% CI	Mean	95% CI	Mean	95% CI	Control	Probiotics	Synbiotics
Energy (kcal)	316.6	(−29.6, 662.7)	−76.4	(−654.9, 502.0)	274.3	(44.7, 504.0)	.07	.79	.02
Weight (g)	397.3	(−12.6, 807.2)	14.3	(−689.0, 717.7)	84.1	(−356.8, 525.1)	.06	.97	.69
Water (g)	327.0	(−42.0, 696.0)	31.4	(−565.7, 628.4)	28.7	(−371.1, 428.5)	.08	.91	.88
Protein (g)	9.3	(−5.7, 24.3)	−6.8	(−28.5, 14.8)	5.9	(−3.7, 15.5)	.21	.51	.21
Lipid (g)	6.8	(−3.9, 17.5)	−5.5	(−22.0, 11.0)	11.3	(1.7, 20.9)	.19	.49	.02
Carbohydrate (g)	47.6	(−7.9, 103.1)	−7.1	(−101.3, 87.2)	35.8	(−2.2, 73.9)	.09	.88	.06
Fiber (g)	2.0	(−0.3, 4.3)	−1.1	(−4.3, 2.2)	1.6	(−1.7, 4.9)	.08	.50	.31

Means indicate the difference of each intake in responders and non-responders. “Responder” was defined as a subject whose bowel movement frequency increased by 2 and more in the intervention period compared with the observation period. CI = confidence interval. *P* values were calculated with Student *t* test.

## 4 Discussion

This randomized, double-blinded, placebo-controlled trial examined whether a probiotic strain, *Bifidobacterium longum* NT, improves constipation, and whether any improvement of constipation was further enhanced by the addition of a prebiotic, galactooligosaccharide, to the *Bifidobacterium longum* NT strain probiotic in healthy adults with constipation tendency. Approximately 90% of participants in this study were women. This could partially be owing to the prevalence of constipation in women being more than double that in men.^[2]^ Bowel movement frequency was the primary outcome in evaluation of whether interventions improved constipation symptoms, and there was no improvement over a placebo control by either intervention. Of the secondary outcomes, stool symptoms in defecation were improved by the probiotic food compared with the placebo control, but they were not improved by the synbiotic food compared with the placebo control (Tables 4 and 5). Abdominal and rectal symptoms were not improved by either intervention. Meanwhile, according to defecation records, the degree of incomplete evacuation was improved by the probiotic food compared with the placebo control (Table 4), and it also tended to be improved in PAC-SYM (Table 5). The use of probiotic *Bifidobacterium longum* NT strain was indicated by this study to improve constipation by affecting the stool condition, which may affect incomplete defecation. There was no enhancement of improvement of constipation with addition of galactooligosaccharide to *Bifidobacterium longum* NT strain.

Bowel movement frequency in the second week of the intervention period was not increased in the probiotics or synbiotics groups compared with the control group. It tended, however, to be time-dependently increased in the control group as well as in the probiotics group. Meta-analysis indicated that a placebo increased bowel movement in patients with chronic idiopathic constipation.^[23]^ Another clinical trial also indicated that a placebo increased bowel movement in healthy adults with constipation tendency.^[19]^ The time-dependent increase of bowel movement frequency may therefore be associated with placebo effects. To further explore the factors that increased bowel movement frequency in the control group, we focused on the difference in dietary history between responders and non-responders in the control group, because intake of fiber and water promote peristaltic movement and softer stools.^[22,24]^ BDHQ indicated that in the control group, responders tended to have higher intake of food (energy and volume), fiber and water than non-responders. In the synbiotics group, responders consumed more total energy and lipid than non-responders (Table 6). These tendencies were not observed in the probiotics group. Although no significant increase in frequency of bowel movement was observed in probiotics compared with the placebo control and synbiotics groups, *Bifidobacterium longum* NT strain may increase bowel movement frequency if the bias, dietary conditions affecting constipation, could be excluded.

Functional constipation in Rome IV criteria includes the symptoms of straining, hard stools, and the feeling of incomplete defecation.^[25]^ It may be difficult to clearly see the effect of probiotics and synbiotics foods on constipation symptoms in healthy men and women with constipation tendency because they have milder these symptoms than the patients with constipation. A clinical trial indicated, however, that a dose of *Bifidobacterium coagulans* lilac-01 improved constipation symptoms in patients with constipation, but not in healthy adults with constipation tendency. On the other hand, another clinical trial indicated that feeling of incomplete defecation in healthy subjects was ameliorated by *B. coagulans* SANK 70258, similarly to in this current study.^[20]^ Constipation symptoms were indicated by this study to be improved by consumption of probiotic food. Thus, probiotic foods may improve constipation symptoms in not only patients with constipation, but also in healthy adults with constipation tendency.

Probiotic *Bifidobacterium longum* NT strain improved incomplete defecation according to patient records of defecation (*P* = .04), it tended to improve incomplete defecation according to PAC-SYM (*P* = .08), which also showed improved stool symptoms (*P* = .02). This study evaluated the stool symptoms in defecation records and PAC-SYM. PAC-SYM results indicate the trends of 12 kinds of constipation symptoms in each period. The reported symptoms in PAC-SYM might, however, be better reflected in the latter half of each period than in the earlier half of each period comparing defecation records in which defecation symptoms were recorded just after defecation because PAC-SYM was answered on last day of each period. The use of probiotics might therefore improve incomplete defecation in the earlier half of the intervention period and decrease intestinal transit time followed by improvement of the stool condition. Previous randomized, double-blinded, placebo-controlled trials demonstrated that the consumption of *Bifidobacterium* (from 1.0 × 10^9^ to 1.5 × 10^10^ CFU/d) for 4 weeks did not improve overall scores on constipation symptoms in PAC-SYM.^[26,27]^ The current study, meanwhile, demonstrated that consumption of *Bifidobacterium longum* NT strain for 2 weeks improved overall scores concerning constipation symptoms in PAC-SYM, although there was no improvement of bowel movement frequency. The *Bifidobacterium longum* NT strain is therefore not inferior to other strains of *Bifidobacterium* in improving symptoms of constipation.

Prebiotics promote the proliferation of probiotics in the colon; they are oligosaccharides, which are non-digestive food components.^[28]^ Galactooligosaccharide included as prebiotics promote the proliferation of *Bifidobacterium*, and the number of *Bifidobacterium* are increased in the feces of healthy adults.^[29]^ Synbiotics are the combined use of prebiotics with probiotics to enhance beneficial effects induced by probiotics. Several clinical trials have demonstrated increased bowel movement frequency by synbiotics compared with placebo controls, but not compared with probiotics.^[12,28]^ Whether the increase of bowel movement frequency is caused by the addition of prebiotics to probiotics, or if this effect is due to probiotics alone has been unclear. The current study confirmed the increase of frequency of bowel movement by administration of probiotics, and examined whether this effect of probiotics was enhanced by the additional administration of prebiotics. A previous prebiotics study indicated that the dose of galactooligosaccharide (5.0 g/day) increased the volume of *Bifidobacterium* in stools compared with other doses (2.5, 7.5, and 10.0 g/day).^[30]^ Meanwhile, another prebiotics study showed that the dose of galactooligosaccharide (2.5 g/day) led to greater improvement than the dose of galactooligosaccharide (5.0 g/day).^[31]^ The use of synbiotics could therefore lead to excessive *Bifidobacterium* in the stool due to additional *Bifidobacterium* and galactooligosaccharide and not from improvement of constipation if the dose of galactooligosaccharide in synbiotics food was 2.5 g/day. We determined the dose of galactooligosaccharide in symbiotic food as 1.0 g/day, which is approximately half of the lower dose in previous study.^[31]^ This was based on a previous study which used the dose of *Bifidobacterium lactis* GCL2505 (10^10^ CFU/day) and which indicated that the amount of *Bifidobacterium lactis* in feces was approximately doubled by probiotic food.^[19]^ We therefore determined that the dose of *Bifidobacterium longum* NT strain in symbiotic and probiotic foods as 10^10^ CFU/day.

Probiotics did not increase bowel movement frequency, but they did improve symptoms related to constipation. Further improvement of constipation symptoms was expected from the use of synbiotics, but such improvement was not observed in this study (Tables 4 and 5). To explore why synbiotics did not further improve constipation symptoms induced by probiotics, we focused on the possibility that *Bifidobacterium longum* NT strain may not actually be increased in the colon as a result of taking galactooligosaccharide. Galactooligosaccharide time-dependently increases *Bifidobacterium* in human fecal microflora.^[11,12]^ A previous study indicated that galactooligosaccharide increased *Bifidobacterium* in human fecal microflora for more than 14 days, but a synbiotic combination of galactooligosaccharide and *Bifidobacterium lactis* HN019 needed 28 days to increase it.^[11]^ The administration of our synbiotic food for 14 days might not therefore increase *Bifidobacterium* as expected in the colon, and it might result in symptoms related to non-improvement of constipation. In another possibility, galactooligosaccharide could remarkably promote the proliferation in *Bifidobacterium longum* NT strain. A clinical trial indicated that the dose of *Bifidobacterium coagulans* lilac-01 (10^8^ CFU/day) improved constipation symptoms in patients with constipation.^[20]^ Another study indicated that the dose of *Bifidobacterium animalis subsp. lactis HN019* increased bowel movement frequency in low (10^9^ CFU/day) and high (10^10^ CFU/day) dose groups in patients with constipation.^[27]^ Stool frequency significantly improved in a low-dose *Bifidobacterium* group (10^9^ CFU/day), but the improvement was attenuated by high-dose of *Bifidobacterium* (10^10^ CFU/day).^[32]^ Adequate dose of *Bifidobacterium* may therefore change intestinal flora, which improves constipation symptoms. Although stool frequency being dose-dependently associated with the administration of probiotics remains controversial, synbiotics might over-promote the proliferation of *Bifidobacterium longum* NT strain, which in the current study may have led to the reduced improvement of symptoms related to constipation. Synbiotics theories insist that prebiotics enhance the beneficial effects of probiotics and some intestinal flora, suggesting that the beneficial effect of probiotics should be enhanced by any prebiotics. In this study, however, prebiotics attenuated the beneficial effect from probiotics. Some patients with constipation consumed functional food including probiotics and prebiotics as part of self-care for their constipation. This suggests that the excessive consumption of prebiotic or probiotic foods may have the effect of cancelling out improvement of constipation symptoms and in some cases, it may even promote constipation. Patient consumption of prebiotic and probiotics food during consultations for constipation should be given due attention.

This study did not indicate improvement of bowel movement frequency (the primary outcome) by 4-week use of probiotics or synbiotics. The sample size was calculated from bowel movement frequency in a previous study,^[21]^ but the number of participants did not meet the estimated number due to withdrawals. Statistical indication of improvement of bowel movement frequency could not therefore be achieved in this study. Meta-analysis indicated that probiotic treatment from between 14 and 84 days also increased stool frequency in healthy adults and in patients with functional constipation.^[8]^ The probiotic foods in this study may therefore have needed an intervention period longer than 14 days to improve bowel movement frequency. In this study, we did not analyze the gut microbial flora in participant's stools, and galactooligosaccharide may unexpectedly inhibit or remarkably promote the proliferation of *Bifidobacterium longum* NT strain in the colons of synbiotics group participants. We collected stool samples to confirm stool conditions, but the next retrospective study will be based on analysis of gut microbial flora in stools to examine whether galactooligosaccharide promotes the proliferation of *Bifidobacterium longum* NT strain.

We showed the effect of synbiotics on constipation in comparison with a placebo control and probiotics food by itself, based on the hypothesis that prebiotics promote the effect of probiotics on constipation. The synbiotic food, the combination of *Bifidobacterium longum* NT strain and galactooligosaccharide, did not improve constipation. It is unclear whether the combination of this strain and other oligosaccharides would improve constipation.

In conclusion, probiotic *Bifidobacterium longum* NT strain (10^10^ CFU/day) did not increase bowel movement frequency, but it improved the symptoms of constipation, especially the condition of stools. The synbiotic *Bifidobacterium longum* NT strain with galactooligosaccharide unexpectedly did not enhance the improvement of constipation that had been shown by probiotic *Bifidobacterium longum* NT strain alone. The addition of prebiotics to probiotics does not necessarily enhance the beneficial effect from probiotics, and it may even attenuate the beneficial effect in some cases.

## Acknowledgments

We acknowledge editing and proofreading by Benjamin Phillis from the Clinical Study Support Center at Wakayama Medical University.

## Author contributions

**Conceptualization:** Yuta Yamamoto, Takao Maekita, Masayuki Kitano.

**Formal analysis:** Takanori Yoshikawa, Kensuke Tanioka.

**Funding acquisition:** Yuta Yamamoto.

**Investigation:** Daisaku Ito, Yuta Yamamoto, Takao Maekita, Naoko Yamagishi, Shuji Kawashima, Takeichi Yoshida, Mikitaka Iguchi, Kosei Kunitatsu, Masayuki Kitano.

**Methodology:** Yuta Yamamoto, Kensuke Tanioka.

**Project administration:** Yuta Yamamoto.

**Supervision:** Yuta Yamamoto, Takao Maekita, Yoshimitsu Kanai, Seiya Kato, Masayuki Kitano.

**Writing – original draft:** Daisaku Ito, Yuta Yamamoto, Takao Maekita, Takeichi Yoshida.

## Supplementary Material

Supplemental Digital Content

## Supplementary Material

Supplemental Digital Content

## Supplementary Material

Supplemental Digital Content
