# Supplementary material for: Do synbiotics really enhance beneficial synbiotics effect on defecation symptoms in healthy adults? Randomized, double-blind, placebo-controlled trial
Source: Medicine (Baltimore). 2022 Feb 25;101(8):e28858. doi: 10.1097/MD.0000000000028858 (PMC8878738; doi:10.1097/MD.0000000000028858)
Supplement: Supplemental Digital Content [file medi-101-e28858-s001.docx]

Supplemental Digital Content 1. Bristol stool scale table.

Supplemental Digital Content 1 Bristol stool scale.

| Type |  |
| --- | --- |
| 1 | Separate hard lumps, like nuts (hard to pass) |
| 2 | Sausage-shaped but with cracks on its surface |
| 3 | Like a sausage but with cracks on its surface |
| 4 | Like a sausage or snake, smooth and soft |
| 5 | Soft blobs with clear-cut edges (passed easily) |
| 6 | Fluffy pieces with ragged edges, a mushy stool |
| 7 | Watery, no solid pieces. Entirely Liquid |

Bristol scale is an indicator of stool hardness and shapes. Smaller numbers of stool type indicate hard and little water in stool, and larger numbers indicate soft or watery stool.
