# Supplementary material for: Do synbiotics really enhance beneficial synbiotics effect on defecation symptoms in healthy adults? Randomized, double-blind, placebo-controlled trial
Source: Medicine (Baltimore). 2022 Feb 25;101(8):e28858. doi: 10.1097/MD.0000000000028858 (PMC8878738; doi:10.1097/MD.0000000000028858)
Supplement: Supplemental Digital Content [file medi-101-e28858-s002.docx]

Supplemental Digital Content 2. PAC-SYM questionnaire table.

Supplemental Digital Content 2 PAC-SYM (The Patient Assessment of Constipation Symptoms) questionnaire.

| How severe have each of these symptoms been in the last 2 weeks? | Absent | Mild | Moderate | Severe | Very Severe |
| --- | --- | --- | --- | --- | --- |
|  | 0 | 1 | 2 | 3 | 4 |
| 1. Discomfort in your stomach |  |  |  |  |  |
| 2. Pain in your stomach |  |  |  |  |  |
| 3. Bloating in your stomach |  |  |  |  |  |
| 4. Stomach cramps |  |  |  |  |  |
| 5. Painful bowel movements |  |  |  |  |  |
| 6. Rectal burning during or after a bowel movement |  |  |  |  |  |
| 7. Rectal bleeding or tearing during or after a bowel movement |  |  |  |  |  |
| 8. Incomplete bowel movement, like you did not “finish” |  |  |  |  |  |
| 9. Bowel movements that were too hard |  |  |  |  |  |
| 10. Bowel movements that were too small |  |  |  |  |  |
| 11. Straining or squeezing to try to pass bowel movements |  |  |  |  |  |
| 12. Feeling like you had to pass a bowel  movement but you could not (“false alarm”) |  |  |  |  |  |

PAC-SYM is questionnaires consisting 12 questions about abdominal, rectal and stool symptoms (abdominal symptoms, 1-4; rectal symptoms, 5-7; and stool symptoms, 8-12). PAC-SYM = Patient Assessment of Constipation Symptoms.
