# Supplementary material for: Do synbiotics really enhance beneficial synbiotics effect on defecation symptoms in healthy adults? Randomized, double-blind, placebo-controlled trial
Source: Medicine (Baltimore). 2022 Feb 25;101(8):e28858. doi: 10.1097/MD.0000000000028858 (PMC8878738; doi:10.1097/MD.0000000000028858)
Supplement: Supplemental Digital Content [file medi-101-e28858-s003.docx]

Supplemental Digital Content 3. Statistical analysis methods.

| Outcomes | Detail | Statistical analysis |
| --- | --- | --- |
| **Primary outcome** |  |  |
| Bowel movement frequency in the 4^th^ week | The difference of bowel movement frequency in the 4^th^ week | Tukey-HSD |
| **Secondary outcomes** |  |  |
| Bowel movement frequency in the 1^st^, 2^nd^, or 3rd week. | The difference of bowel movement frequency in the 1^st^, 2^nd^, or 3^rd^ week. | Tukey-HSD |
| Bowel movement frequency in the observation or intervention periods. | The difference of bowel movement frequency between the observation and intervention periods. | Dunnett |
| Number of days with defecation in the observation or intervention periods. | The difference of number of days with defecation between the observation and intervention periods. | Dunnett |
| Feeling discomfort (straining, pain, and incomplete defecation) at defecation in the observation or intervention periods. | The difference in average degree of discomfort at defecation between the observation and intervention periods. | Dunnett |
| Bristol stool scale score at defecation in the observation/intervention periods. | The difference in average of Bristol stool scale score between the observation period and intervention period | Dunnett |
| PAC-SYM score (12 questions, abdominal (1-4), rectal (5-7), and stool symptom (8-12), and total score) in the observation/intervention periods. | The difference in PAC-SYM scores between observation and intervention periods | Dunnett |
| Nutrition intake (energy, food weight, water, protein, lipid, carbohydrate, and fiber) in observation/intervention periods. | The difference in nutrition intake between responders and non-responders. | Student’s t |

Tukey-HSD test compares the means of every group with the means of every other group. Dunnett’s test compares the means of each group (probiotics or synbiotics group) with the means of the control group. Student’s t test compares the means of the responders to the means of non-responders. Number of days with defecation was the number of days in which a subject defecates at least once per day.
